# Risk factors for avascular necrosis after closed reduction for developmental dysplasia of the hip

**DOI:** 10.1007/s11832-016-0743-7

**Published:** 2016-05-13

**Authors:** Mathew D. Schur, Christopher Lee, Alexandre Arkader, Anthony Catalano, Paul D. Choi

**Affiliations:** Children’s Orthopaedic Center, Children’s Hospital Los Angeles, 4650 Sunset Boulevard, Mailstop #69, Los Angeles, CA 90027 USA; Department of Orthopaedic Surgery, University of California Los Angeles, Los Angeles, CA USA; Department of Orthopaedic Surgery, Children’s Hospital of Philadelphia, Philadelphia, PA USA

**Keywords:** Avascular necrosis, Developmental dysplasia of the hip, Closed reduction, Abduction angle

## Abstract

**Purpose:**

The purpose of this study was to identify and evaluate risk factors of avascular necrosis (AVN) after closed treatment for developmental dysplasia of the hip (DDH).

**Methods:**

A retrospective review of children diagnosed with DDH at a tertiary-care children’s hospital between 1986 and 2009 was performed. The presence of AVN was assessed according to Salter’s classification system.

**Results:**

Eighty-two affected hips in 70 children with an average age of 10 months at closed reduction (range 1–31 months) and 5 years (range 2–19 years) of follow-up met the inclusion criteria. Twenty-nine (of 82, 35 %) affected hips developed AVN. The use of pre-reduction traction (*p* = 0.019) increased the risk of AVN, while preoperative Pavlik harness or brace trial (*p* = 0.28), presence of ossific nucleus at the time of closed reduction (*p* = 0.16), and adductor tenotomy (*p* = 0.37) were not significant factors. Laterality (right vs. left) was also not a significant risk factor (*p* = 0.75), but patients who underwent closed reduction for bilateral DDH were less likely to develop AVN (*p* = 0.027). Overall, the degree of abduction did not affect the rate of AVN (*p* = 0.87). However, in patients treated with closed reduction younger than 6 months of age, the rate of AVN was increased with abduction ≥50° (9/15, 60 %) compared to abduction <50° (0/8, 0 %) (*p* = 0.007). Patients who developed AVN were more likely to require subsequent surgery (*p* = 0.034) and more likely to report a fair/poor clinical outcome (*p* = 0.049).

**Conclusions:**

The risk of AVN (35 %) following closed reduction and spica casting for DDH is high. The degree of abduction in spica casts appears to be a risk factor in patients ≤6 months old. The authors recommend that abduction in spica casts should be limited to <50° in children younger than 6 months of age.

**Level of evidence:**

IV.

## Introduction

Developmental dysplasia of the hip (DDH) has an estimated incidence of 1.5–20 per 1000 births [1]. Closed reduction plus spica cast application has a success rate as high as 95 %, but complications do occur [2–10]. Complication rates have been reported as high as 79 %, and avascular necrosis (AVN) has proven to be particularly problematic [3, 5, 10–15]. This major complication has a reported incidence of up to 47 % and can result in limb length discrepancy, joint incongruity, persistent subluxation, coxa valga, and other sequelae [6, 8, 16–24]. These changes can drastically affect hip function and may impact overall health and quality of life [18, 22–26].

The exact etiology of AVN is unknown but likely multifactorial, with vascular and iatrogenic components [18, 26–28]. Prior literature identifies both a number of risk factors, such as hip abduction angle, and protective factors, such as early age at reduction, the presence of an ossific nucleus, and the use of an adductor tenotomy in the development of AVN [18–20, 22–25, 29–31]. Although some of these factors have been previously analyzed, the significance of each in the development of AVN has remained unclear. The purpose of this study was to identify and evaluate the risk factors and outcomes associated with AVN after closed reduction and spica cast application for children with DDH.

## Materials and methods

After institutional review board approval, initial screening using International Classification of Diseases, Ninth Revision, Clinical Modification (ICD-9-CM) codes for the diagnosis of DDH and Current Procedural Terminology (CPT) codes for closed reduction between January 1986 and January 2009 identified 287 consecutive patients at a single tertiary pediatric center. Inclusion criteria were a diagnosis of a subluxated or dislocated hip requiring closed reduction under general anesthesia in the operating room plus spica cast application, computed tomography (CT) scan following reduction, and a minimum of 18 months of clinical and radiographic follow-up. Exclusion criteria were a diagnosis of neuromuscular disease or teratologic dislocations, history of open reduction as initial management, incomplete radiographic data, history of multiple closed reductions, and onset of AVN after subsequent surgery.

Retrospective electronic and physical chart reviews were performed to identify demographic information, clinical data, and length of follow-up. The need for subsequent procedures, whether open reduction of the hip or a reconstructive procedure (acetabular augmentation and/or femoral osteotomy), was also recorded. Images were reviewed by a single observer not involved in patient treatment. The initial preoperative plain radiographs were reviewed for the presence of a proximal femoral ossific nucleus. CT scans following closed reduction and spica casting were reviewed to measure the hip abduction angle by the method described by Browning et al. [32]. Plain radiographs at 1 year and final follow-up were reviewed to look for the development of AVN and to measure the acetabular index. The presence of AVN was evaluated using Salter’s criteria [18]. Temporary irregular ossification, as defined by Salter et al., was not considered AVN [18]. Clinical outcomes were graded as excellent, good, fair, or poor based on the criteria devised by Brougham et al. [22]. Radiographic outcomes were graded based on the acetabular index, which was measured manually. Acetabular dysplasia was defined by an acetabular index ≥30° in the affected hip at 2 years of age [33].

Statistical analysis was performed using Stata 12 (StataCorp LP, College Station, TX) and Microsoft Excel (2010). Univariate statistical analysis was performed by two-tailed Student’s *t*-test and the Chi-squared test or Fisher’ exact test in the case that values were too small for Chi-squared. A general estimating equation (GEE) was used to analyze the association of AVN with multiple variables while accounting for the cases of bilateral affected hips. Logistic multivariate regression was used for subgroup (closed reduction within six months of age) analysis, as the population was too small to use a GEE. Variables were included in either multivariate model if the univariate *p*-value <0.15 [34]. *p*-Value were considered significant at *p* < 0.05. Univariate *p*-values are given for variables that did not meet the multivariate inclusion threshold (*p* < 0.15). Effect sizes are reported as odds ratio (OR) or risk difference (RD) when unable to calculate the OR due to a zero being included in the calculation. Figures were created using Adobe Photoshop CS6 (Adobe Systems, San Jose, CA).

## Results

Seventy patients with 82 hips underwent closed reduction under general anesthesia and spica cast application for a subluxated or dislocated hip and met the inclusion criteria. Sixty-two of these patients (89 %) were female and eight (11 %) were male. The left hip was involved in 40 patients, the right in 18 patients, and both hips in 12 patients. The mean age at the time of diagnosis was 8.0 months (range 0–31 months) and the mean age at the time of closed reduction was 10 months (range 1–31 months). The mean length of follow-up was 5 years (range 2–19 years).

Twenty-two (of 70, 31 %) had undergone Pavlik harness or hip abduction orthosis treatment prior to the closed reduction procedure under general anesthesia (duration 1–12 weeks) (Table 1). In general, Pavlik harness or hip abduction orthosis was used as the initial treatment for a subluxated or dislocated hip in younger patients (younger than 6–9 months of age). Traction was used in 17 patients (of 70, 24 %) for 3–14 days at weights ranging from 1 to 10 pounds (11–38 % body weight traction), with ages ranging from 2 to 15 months. Three types of traction were used: Bradford traction (longitudinal type of traction), Bryant traction (overhead traction), and gallows-type traction (also overhead). The proximal femoral ossific nucleus was present in 48 (of 82, 59 %) affected hips on plain radiographs at the time of the closed reduction procedure. Fifty-two patients (of 70, 74 %) underwent adductor tenotomy during the closed reduction procedure under general anesthesia prior to spica cast application.

**Table 1:** Preoperative or intraoperative treatments

Treatment	Number of patients	Number of hips
Pavlik harness or abduction brace	22 (1–12 weeks)	29
Bradford/Bryant/gallows-type traction	17 (3–14 days)	21
Adductor tenotomy	52	62

AVN of the femoral head developed in 29 of 82 affected hips (35 %). AVN developed in 28 of 70 patients (40 %). Twenty-one of these patients (75 %) were female and seven (25 %) were male. The left hip was involved in 18 patients, the right in nine, and both hips in one patient. Male patients were significantly more likely to develop AVN compared to female patients (*p* = 0.027). Patients with bilateral DDH were less likely to develop AVN (*p* = 0.027), while laterality (right vs. left) did not appear to affect the risk of AVN (*p* = 0.85).

No significant difference in the development of AVN was seen whether patients had undergone prior Pavlik harness or hip abduction orthosis treatment (*p* = 0.28) or whether adductor tenotomy was performed during the closed reduction procedure (*p* = 0.37). The use of pre-reduction traction appeared to increase the risk of AVN, with AVN developing in 65 % (11/17) of patients who underwent traction versus 32 % (17/53) who did not (*p* = 0.019). There was no significant difference in the rates of AVN in patients who underwent longitudinal versus overhead (vertical) traction (*p* = 0.71). Four of the 17 patients (five affected hips, one bilateral case) that underwent pre-reduction traction had undergone and failed brace treatment (Pavlik harness or hip abduction orthosis). A prior history of brace treatment did not affect the risk of AVN in patients who underwent pre-reduction traction; the risk of AVN remained increased. In pre-reduction patients with history of brace treatment, three (of five) hips (in two of four patients) developed AVN, while in pre-reduction patients without history of brace treatment, nine (of 16) hips (in 9 of 13 patients) developed AVN (*p* > 0.99).

**Fig. 1:**
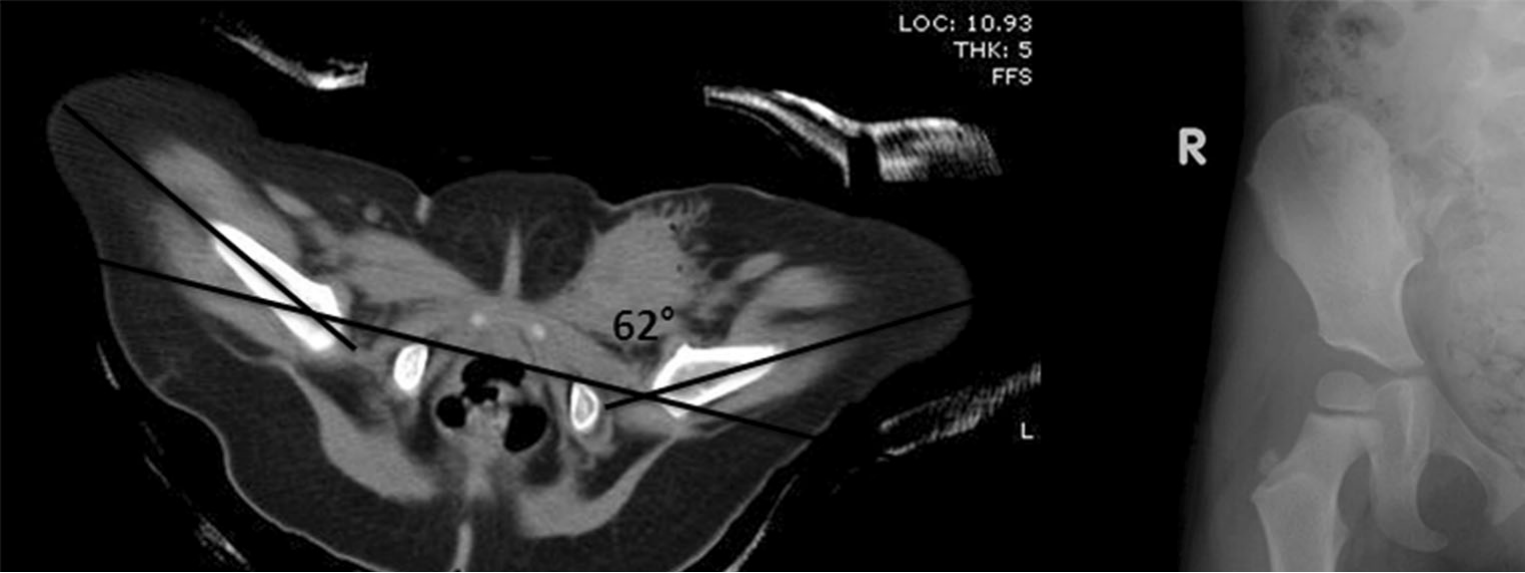
Five-month-old female patient diagnosed with developmental dysplasia of the left hip. The patient underwent failed pre-reduction Pavlik harness treatment for eight days and did not have ossific nuclei present at the time of closed reduction. **a** Following an adductor tenotomy, closed reduction, and spica cast application, postoperative computed tomography (CT) revealed the left hip to be abducted to 62°. **b** Radiographs at 1-year follow-up indicate deformity of the left hip indicative of avascular necrosis (AVN). The patient’s final clinical score was graded as fair

No significant difference in the risk of AVN was seen whether proximal femoral ossific nucleus was present or not at the time of closed reduction (*p* = 0.16). The hip abduction angle also did not appear to affect the risk of AVN (*p* = 0.87) in the overall study population. However, in patients ≤6 months of age at the time of the closed reduction procedure, hip abduction angle ≥50° did significantly increase the risk of AVN [*p* = 0.007, RD 0.6, 95 % confidence interval (CI) 0.19–0.80] (Fig. 1). The proximal femoral ossific nucleus was present in six (of 23) hips that underwent closed reduction younger than 6 months of age; 42 (of 59) hips that underwent closed reduction were older than 6 months. The risk of AVN remained increased in this group of patients ≤6 months of age at the time of closed reduction and hip abduction angle ≥50° whether proximal femoral ossific nucleus was present or not. In patients younger than 6 months old and hip abduction angle ≥50°, eight (of 12) hips (in seven of 11 patients) developed AVN when the ossific nucleus was absent, compared to one (of three) hips (in one of three patients) when the ossific nucleus was present (*p* = 0.53). Furthermore, in patients without ossific nuclei (29 of 70 patients), hip abduction angle ≥50° did not significantly increase the risk of AVN (*p* = 0.29). The results of univariate and multivariate analyses to determine the significance of risk factors of AVN (including effect sizes and confidence intervals) can be found in Table 2.

**Table 2:** Risk factors of avascular necrosis (AVN): results of univariate and multivariate analyses to determine significance

Risk factors	Univariate *p*-value	Odds ratio	95 % CI	Multivariate *p*-value	Odds ratio	95 % CI
Male gender	0.011	16.6	1.92–142.58	0.027	16.2	1.38–190.76
Age at diagnosis	0.28	1.0	0.97–1.13			
Bilateral	0.06	0.3	0.11–1.05	0.027	0.2	0.03–0.82
Left side	0.85	0.9	0.36–2.33			
Age >6 months at CR	0.66	0.8	0.29–2.16			
Ossific nucleus present at CR	0.16	0.5	0.21–1.31			
Bracing before CR	0.28	0.6	0.22–1.55			
Traction before CR	0.018	3.5	1.23–9.66	0.019	5.8	1.34–25.43
Adductor tenotomy	0.11	2.7	0.81–9.04	0.37	2.2	0.39–12.70
Hip abduction angle	0.87	1.0	0.97–1.04			
Hip abduction angle ≥50°	0.57	1.4	0.47–4.08			

*CR* closed reduction, *CI* confidence interval, *Bracing* Pavlik harness/hip abduction orthosis

Variables included in the multivariate model if univariate *p*-value <0.15 [34]

### Outcome

Sixteen of 70 patients (23 %) underwent subsequent surgery, with 13 acetabular augmentation procedures (Salter or Dega pelvic osteotomy), one femoral osteotomy, one acetabular augmentation + femoral osteotomy + open reduction, and one open reduction. Patients who developed AVN were significantly more likely to require subsequent surgery (*p* = 0.034). Ten (of 28, 36 %) of the patients who developed AVN after the closed reduction procedure ultimately required subsequent surgery (eight acetabular augmentation, one acetabular augmentation + femoral osteotomy + open reduction, one open reduction), compared to six (of 42, 14 %) of the patients without AVN (five acetabular augmentation, one femoral osteotomy) (Table 3). Overall, of the 82 affected hips, 71 were clinically graded as good to excellent (55 excellent, 16 good), while 11 hips were graded as fair to poor (11 fair, 0 poor). Patients with AVN were significantly more likely to report fair to poor clinical outcome (8/29) than patients without AVN (3/53) (*p* = 0.049) (Table 4). The mean acetabular index of affected hips at final follow-up was 19.4° (range 2°–36°). The development of AVN did not result in a higher acetabular index (mean acetabular index in AVN patients = 20.0°; in non-AVN patients = 19.1°) (*p* = 0.59). The results of the univariate and multivariate analyses to determine the relationship between AVN and clinical and radiographic outcomes can be found in Table 5.

**Table 3:** Additional surgeries required after initial closed reduction

Additional surgery	Number of patients	
	With AVN	Without AVN
Acetabular augmentation	8	5
Femoral osteotomy	0	1
Acetabular augmentation + femoral osteotomy + open reduction	1	0
Open reduction	1	0

**Table 4:** Clinical outcome of patients with and without AVN graded on the scale by Brougham et al. [22]

Clinical outcome	Number of patients	
	With AVN	Without AVN
Excellent—painless, no limp, negative Trendelenburg, combined ROM >300°	13	42
Good—painless, normal gait or slight limp, negative or delayed positive Trendelenburg, combined ROM <300° and >200°	8	8
Fair—painless or occasional ache, limp and/or Trendelenburg sign and/or combined ROM >150° and <200°	8	3
Poor—pain (exercise-related or interfering with function), positive Trendelenburg and/or fixed deformity and/or combined ROM <150°	0	0

**Table 5:** Relationship between AVN and clinical and radiographic outcomes: results of the univariate and multivariate analyses

Outcomes	Univariate *p*-value	Odds ratio	95 % CI	Multivariate *p*-value	Odds ratio	95 % CI
Acetabular index angle at final follow-up	0.59	1.0	0.95–1.09			
Center edge angle at final follow-up	0.11	1.0	0.93–1.01	0.13	1.0	0.88–1.02
Excellent/good vs. fair/poor clinical outcome	0.011	0.2	0.38–0.65	0.049	0.2	0.04–0.99
Additional surgery required	0.06	2.6	0.95–7.27	0.034	4.6	1.12–18.62

Variables included in the multivariate model if univariate *p*-value <0.15 [34]

## Discussion

AVN of the femoral head is a known complication following closed reduction and spica cast application for DDH (a subluxated or dislocated hip), with rates reported as high as 47 % [22]. Earlier studies have cited various possible risk factors of AVN, including age, gender, laterality, absence of proximal femoral ossific nucleus, use of pre-reduction traction, preliminary/history of hip abduction bracing, adductor tenotomy, and hip abduction angle in cast [18–20, 22–25, 29–31]. Brougham et al. reported on 184 patients who underwent closed reduction of 210 hip dislocations and noted AVN in 99 (of 210 affected hips, 47 %) [22]. Risk of AVN was unaffected by age, gender, laterality, previous use of hip abduction orthosis, or adductor tenotomy. Sibiński et al. reported AVN in 36 % of DDH hips (37 of 103 affected hips) treated with closed reduction and cast immobilization [35]. Laterality, absence of proximal femoral ossific nucleus, use of pre-reduction traction, and hip abduction bracing did not affect the AVN risk. However, the degree of initial dislocation (by the Tönnis classification) and age at onset of treatment were significant risk factors of AVN. Carney et al., in their study of 45 patients who underwent successful closed reduction of a DDH hip, noted AVN in 17 (of 48 affected hips, 35 %) and reported the presence of the proximal femoral ossific nucleus and use of adductor tenotomy as significant protective factors resulting in lower risk/rate of AVN [36]. In a study of 64 patients with developmental dislocation of the hip (36 who underwent closed reduction, 28 who were treated with open reduction), Pospischill et al. reported AVN in 40 % of hips (31 of 78 hips) and identified open reduction combined with concomitant osteotomies, redislocation, and need for secondary procedures after initial reduction as risk factors of AVN. Age, preliminary traction, and spica cast immobilization were not found to increase the risk of AVN [24].

In this series of 82 hips, the incidence of AVN was 35 % (29 of 82 affected hips). Similar to previous studies, a large number of risk factors for AVN after closed reduction and spica casting for a dislocated or subluxated hip were comprehensively reviewed. One specific focus of the study was the effect of hip abduction angle on the risk of AVN, in particular, whether the effects of hip abduction angle occur in an age-dependent manner.

In this series, gender was found to be a significant risk factor of AVN, with seven (of eight, 88 %) affected hips in males compared to 21 (of 62, 34 %) hips in females developing AVN (*p* = 0.027). A trend toward higher risk of AVN in male patients has been previously reported; however, it is unclear why male patients would be at higher risk [22, 37]. Similar to previous studies, laterality (right vs. left) in this series was not a significant risk factor of AVN (*p* = 0.85) [22, 35]. But unlike previous studies, this study revealed a decreased likelihood of developing AVN in patients undergoing treatment for bilateral DDH (*p* = 0.027). Morbi et al. found an increased risk of AVN in patients treated for bilateral DDH (33.3 %, 24 of 72 hips) compared to unilateral cases (11.2 %, 25 of 224 hips) [38]. Previous studies (like that of Morbi et al.) reporting an increased risk of AVN for bilateral DDH, however, have included patients treated with (closed and) open reduction [38, 39]. The true effect of bilaterality on the risk of AVN is not clear from this study, as it was limited to patients undergoing closed reduction alone and excluded patients treated with open reduction.

The use of pre-reduction traction was found to be significantly associated with the development of AVN, with an increased rate of AVN in patients who had undergone pre-reduction traction (65 vs. 32 %) (*p* = 0.019). The higher risk of AVN in patients who underwent pre-reduction traction conflicts with traditional thinking that traction can decrease the risk of AVN, as reported in multiple studies [12, 28, 40, 41]. These multiple studies explain that pre-reduction traction can decrease the need for open reduction and, thereby, reduce the risk of AVN. The exact relationship between pre-reduction traction and AVN, however, is unclear, as other studies have not found a decreased rate of open reduction or AVN with pre-reduction traction [8, 22, 24, 35, 42, 43]. Subgroup analysis was performed to determine whether history of prior brace treatment (Pavlik harness or hip abduction orthosis) influenced the role of pre-reduction traction and risk of AVN. Four of the 17 patients (five affected hips, one bilateral case) who underwent pre-reduction traction had history of failed brace treatment. The risk of AVN remained increased in patients who had undergone pre-reduction traction—whether they had history of prior brace treatment or not (*p* > 0.99). The risk of AVN in pre-reduction traction patients was also increased regardless of the type of traction (longitudinal vs. overhead-vertical) (*p* = 0.71).

Similar to other studies, the risk of AVN in this series was not affected by: presence of proximal femoral ossific nucleus, preliminary/history of hip abduction bracing, and adductor tenotomy [22–24, 35, 37, 44]. Upon initial analysis, hip abduction in the spica cast did not appear to affect the risk of AVN (*p* = 0.87): mean abduction angle of 57.3° (37–81) in hips without AVN compared to 57.7° (36–82) in hips that developed AVN. However, upon closer inspection of younger patients (less than or equal to 6 months of age at the time of the closed reduction procedure), abduction in the spica cast greater than or equal to 50° did significantly increase the risk of AVN (*p* = 0.007). Further analysis was performed to explore whether it was age and/or the presence of ossific nucleus that influenced this higher risk of AVN with high hip abduction angle. The risk of AVN remained increased in younger patients with hip abduction angle greater than 50° whether proximal femoral ossific nucleus was present or not (*p* = 0.53). Also, in all patients without proximal femoral ossific nuclei, hip abduction angle ≥50° did not increase the risk of AVN (*p* = 0.29).

Previous literature considering excessive hip abduction as a potential risk factor of AVN lacks a consistent conclusion. Older reports by Salter et al. [18] and Gage and Winter [19] suggest extreme positions of abduction as a potential contributor to the development of AVN. In the years following, this has been both corroborated, in reports from Smith et al. [45] and Gregosiewicz and Wósko [29], and contradicted, as reported by Stanton and Capecci [21]. While our initial analysis of all 70 patients found no significant association between hip abduction and AVN, a more in-depth look identified a positive association in a subset of patients who underwent closed reduction at ≤6 months of age. Also notable was that, within this group, AVN only developed in patients 3–6 months of age at the time of closed reduction. This age-dependent association is consistent with prior clinical, experimental, and anatomical investigations which describe a changing vascular structure mid-way through the first year of life, with significant vulnerabilities to extreme abduction in its transition from bilateral to unilateral supply [18, 23, 27, 46–50]. In the future, a gadolinium-enhanced perfusion magnetic resonance imaging (MRI) after closed reduction/spica casting may help to decrease the risk of AVN by allowing for adjustments in the treatment protocol, including abduction angle in the spica cast, while maintaining perfusion/vascularity of the femoral head [51].

Outcome when AVN develops after closed reduction procedure for DDH can be compromised. The development of AVN increased the likelihood of subsequent surgery (*p* = 0.034), most commonly an acetabular augmentation procedure (13 cases), such as a Salter or Dega pelvic osteotomy. Most commonly, subsequent surgeries were needed because of persistent acetabular dysplasia and/or incomplete reduction. The development of AVN led to poorer clinical grades, with eight (of 29, 28 %) AVN-affected hips reported as fair to poor clinical outcome compared to three (of 53, 6 %) non-AVN hips.

There were several limitations in our study. First, because of its retrospective nature, the study was limited by available patient data. Second, the study period of 23 years was long, resulting in heterogeneity in treatment protocols. Third, with multiple treating surgeons, the role of surgeon skill is unclear and unknown. Finally, radiographic outcome data is subject to rater reliability and image accuracy. Despite the limitations, our study comprehensively examined risk factors for the development of AVN and outcomes following closed reduction and spica casting for DDH in a large patient pool treated at a single tertiary pediatric center. It is also the first to report on the relationship of hip abduction angle and young age on the risk of AVN.

In conclusion, although closed reduction and spica cast application remain an effective and successful treatment option for many patients with DDH, the development of AVN following a closed reduction procedure can be problematic, with our study reporting an incidence of 35 %. On the basis of our study, male gender and the use of pre-reduction traction may increase the risk of AVN. The degree of hip abduction appears to play a significant role in the development of AVN in younger patients (less than or equal to 6 months of age). Based on our results, the authors recommend that abduction in spica casts should be limited to less than 50° in children less than or equal to 6 months of age.
